# Supplementary material for: Variations in HLA-B cell surface expression, half-life and extracellular antigen receptivity
Source: eLife. 2018 Jul 10;7:e34961. doi: 10.7554/eLife.34961 (PMC6039183; doi:10.7554/eLife.34961)
Supplement: Figure 1—source data 1. — HLA class I genotypes of donors used for Bw6 measurements, and mean of ABC values measured with anti-Bw6 and W6/32 for each lymphocyte subset. The HLA-B-Bw6 allele of each donor is highlighted in bold. Standard errors of the mean (SEM) values and the number of replicate measurements (N; with separate blood collections) are indicated. [file elife-34961-fig1-data1.docx]

**Figure 1 - Source Data 1**: **Expression variations among HLA-Bw6 alleles**

HLA class I genotypes of donors used for Bw6 measurements, and mean of ABC values measured with anti-Bw6 and W6/32 for each lymphocyte subset. The HLA-B-Bw6 allele of each donor is highlighted in bold. Standard errors of the mean (SEM) values and the number of replicate measurements (N; with separate blood collections) are indicated.

| Donor ID | Allele 1 | Allele 2 | Cell Type | Bw6 ABC Mean | | | w6/32 ABC Mean | | |
| --- | --- | --- | --- | --- | --- | --- | --- | --- | --- |
|  |  |  |  | Mean | SEM | N | Mean | SEM | N |
| 1 |  |  | B Cell | 141038 | 11953 | 8 | 567841 | 52324 | 7 |
| HLA-A | A*24:02:01:01 | A*29:02:01:01 | CD4+ T Cell | 148817 | 5066 | 8 | 571447 | 51212 | 7 |
| HLA-B | **B*35:01:01** | B*44:03:01 | CD8+ T Cell | 178627 | 8308 | 8 | 716090 | 56098 | 7 |
| HLA-C | C*04:01:01:01 | C*16:01:01 | NK Cell | 190438 | 17951 | 8 | 764518 | 61139 | 7 |
| 5 |  |  | B Cell | 170785 | 11949 | 10 | 581013 | 69160 | 9 |
| HLA-A | A*02:01:01:01 | A*34:02:01 | CD4+ T Cell | 141405 | 5342 | 10 | 571605 | 49465 | 9 |
| HLA-B | **B*35:01:01:02** | B*44:02:01:01 | CD8+ T Cell | 160842 | 9401 | 10 | 679561 | 55661 | 9 |
| HLA-C | C*05:01:01:02 | C*16:01:01 | NK Cell | 154628 | 10356 | 10 | 651834 | 82201 | 9 |
| 8 |  |  | B Cell | 220185 | 16125 | 9 | 584299 | 48069 | 10 |
| HLA-A | A*01:01:01:01 | A*03:01:01:01 | CD4+ T Cell | 208062 | 13801 | 9 | 678588 | 53908 | 10 |
| HLA-B | **B*15:01:01** | B*37:01:01 | CD8+ T Cell | 245282 | 10539 | 9 | 836030 | 57813 | 10 |
| HLA-C | C*01:02:01 | C*06:02:01:01 | NK Cell | 184928 | 13545 | 9 | 706679 | 58151 | 10 |
| 9 |  |  | B Cell | 167446 | 8709 | 10 | 450645 | 30484 | 14 |
| HLA-A | A*01:01:01:01 | - | CD4+ T Cell | 193758 | 8393 | 10 | 532153 | 38765 | 14 |
| HLA-B | **B*08:01:01** | - | CD8+ T Cell | 225455 | 11199 | 10 | 654745 | 47805 | 14 |
| HLA-C | C*07:01:01:01 | - | NK Cell | 204826 | 11801 | 10 | 585297 | 45999 | 14 |
| 12 |  |  | B Cell | 297055 | 18510 | 9 | 761031 | 77301 | 9 |
| HLA-A | A*03:01:01:01 | A*01:01:01:01 | CD4+ T Cell | 203135 | 12343 | 9 | 547346 | 40167 | 9 |
| HLA-B | **B*08:01:01** | B*44:02:01:01 | CD8+ T Cell | 200509 | 10455 | 9 | 577277 | 47404 | 9 |
| HLA-C | C*05:01:01:02 | C*07:01:01:01 | NK Cell | 202498 | 14129 | 9 | 612580 | 59967 | 9 |
| 14 |  |  | B Cell | 208322 | 9109 | 13 | 611082 | 48036 | 21 |
| HLA-A | A*02:01:01:01 | A*03:01:01:01 | CD4+ T Cell | 129698 | 5486 | 13 | 524415 | 31582 | 21 |
| HLA-B | **B*07:02:01** | B*51:01:01:01 | CD8+ T Cell | 200289 | 8195 | 13 | 764337 | 44657 | 21 |
| HLA-C | C*15:02:01:01 | C*07:02:01:03 | NK Cell | 145900 | 7705 | 13 | 599188 | 41974 | 21 |
| 17 |  |  | B Cell | 202935 | 4724 | 5 | 726245 | 174995 | 7 |
| HLA-A | A*03:01:01:01 | A*02:01:01:01 | CD4+ T Cell | 117172 | 4010 | 5 | 459433 | 99370 | 7 |
| HLA-B | **B*07:02:01** | - | CD8+ T Cell | 156131 | 266 | 5 | 623203 | 133077 | 7 |
| HLA-C | C*07:02:01:03 | - | NK Cell | 131471 | 4319 | 5 | 567649 | 110525 | 7 |
| 20 |  |  | B Cell | 255122 | 22740 | 7 | 863865 | 75918 | 12 |
| HLA-A | A*02:01:01:01 | - | CD4+ T Cell | 191963 | 9902 | 7 | 761171 | 66406 | 12 |
| HLA-B | **B*07:02:01** | B*37:01:01 | CD8+ T Cell | 313329 | 15752 | 7 | 1247612 | 104350 | 12 |
| HLA-C | C*07:02:01:03 | C*06:02:01:01 | NK Cell | 180359 | 33997 | 7 | 759299 | 122420 | 12 |

| SAMPLE ID | Allele 1 | Allele 2 | Cell Type | Bw6 ABC Mean | | | w6/32 ABC Mean | | |
| --- | --- | --- | --- | --- | --- | --- | --- | --- | --- |
|  |  |  |  | Mean | SEM | N | Mean | SEM | N |
| 24 |  |  | B Cell | 147561 | 5158 | 15 | 499684 | 54357 | 18 |
| HLA-A | A*02:01:01:01 | A*24:02:01:01 | CD4+ T Cell | 138448 | 6020 | 15 | 502020 | 46670 | 18 |
| HLA-B | **B*35:01:01:02** | B*51:01:01:01 | CD8+ T Cell | 167398 | 6928 | 15 | 627433 | 53901 | 18 |
| HLA-C | C*15:02:01:01 | C*04:04:01 | NK Cell | 125714 | 6104 | 15 | 548666 | 475151 | 18 |
| 26 |  |  | B Cell | 172325 | 11150 | 5 | 742544 | 135273 | 5 |
| HLA-A | A*25:01:01 | - | CD4+ T Cell | 157109 | 15158 | 5 | 673872 | 93578 | 5 |
| HLA-B | **B*18:01:01:02** | - | CD8+ T Cell | 154209 | 12587 | 5 | 715113 | 94608 | 5 |
| HLA-C | C*12:03:01:02 | - | NK Cell | 154065 | 22865 | 5 | 720687 | 115097 | 5 |
| 28 |  |  | B Cell | 185251 | 10354 | 10 | 520365 | 44202 | 14 |
| HLA-A | A*02:01:01:01 | A*01:01:01:01 | CD4+ T Cell | 209579 | 8989 | 10 | 582195 | 51046 | 14 |
| HLA-B | **B*08:01:01** | B*51:01:01:01 | CD8+ T Cell | 224920 | 12940 | 10 | 660475 | 59808 | 14 |
| HLA-C | C*15:13 | C*07:01:01:01 | NK Cell | 222561 | 15571 | 10 | 688130 | 74454 | 14 |
| 31 |  |  | B Cell | 200410 | 8819 | 12 | 649839 | 61506 | 14 |
| HLA-A | A*03:01:01:01 | A*32:01:01 | CD4+ T Cell | 14514 | 5596 | 12 | 577638 | 50329 | 14 |
| HLA-B | **B*07:02:01** | B*44:02:01:01 | CD8+ T Cell | 192410 | 7868 | 12 | 748759 | 64641 | 14 |
| HLA-C | C*07:02:01:03 | C*05:01:01:02 | NK Cell | 154046 | 6046 | 12 | 624094 | 53306 | 14 |
| 34 |  |  | B Cell | 296085 | 35181 | 5 | 765218 | 161285 | 5 |
| HLA-A | A*23:01:01 | A*30:02:01:03 | CD4+ T Cell | 179276 | 25185 | 5 | 522111 | 104566 | 5 |
| HLA-B | **B*18:01:01:01** | B*58:01:01:01 | CD8+ T Cell | 214597 | 16646 | 5 | 651446 | 124480 | 5 |
| HLA-C | C*03:02:02:01 | C*05:01:01:01 | NK Cell | 152799 | 17002 | 5 | 488088 | 113781 | 5 |
| 35 |  |  | B Cell | 308851 | 9459 | 3 | 799739 | 95959 | 5 |
| HLA-A | A*02:01:01:01 | A*01:01:01:01 | CD4+ T Cell | 200321 | 4100 | 3 | 605600 | 87594 | 5 |
| HLA-B | **B*07:02:01** | - | CD8+ T Cell | 239981 | 4419 | 3 | 707899 | 94231 | 5 |
| HLA-C | C*07:02:01:03 | - | NK Cell | 227354 | 6778 | 3 | 685686 | 80944 | 5 |
| 55 |  |  | B Cell | 296597 | 18816 | 13 | 808102 | 87830 | 15 |
| HLA-A | A*23:01:01 | A*01:01:01:01 | CD4+ T Cell | 196628 | 8696 | 13 | 561601 | 47760 | 15 |
| HLA-B | **B*08:01:01** | B*44:03:01 | CD8+ T Cell | 273435 | 12091 | 13 | 778460 | 70546 | 15 |
| HLA-C | C*04:09N | C*07:01:01:01 | NK Cell | 278738 | 13092 | 13 | 778825 | 74032 | 15 |
| 57 |  |  | B Cell | 213369 | 51906 | 3 | 940842 | 109487 | 5 |
| HLA-A | A*02:01:01:01 | A*01:01:01:01 | CD4+ T Cell | 129736 | 24068 | 3 | 654564 | 39119 | 5 |
| HLA-B | **B*07:02:01** | - | CD8+ T Cell | 171311 | 34687 | 3 | 878123 | 57261 | 5 |
| HLA-C | C*07:02:01:03 | - | NK Cell | 160785 | 30811 | 3 | 878766 | 65982 | 5 |
| 64 |  |  | B Cell | 187691 | 12134 | 18 | 69768 | 61970 | 22 |
| HLA-A | A*03:01:01:01 | A*02:01:01:01 | CD4+ T Cell | 141654 | 6830 | 18 | 607467 | 57827 | 22 |
| HLA-B | **B*07:02:01** | B*44:02:01:01 | CD8+ T Cell | 165931 | 8623 | 18 | 699273 | 68952 | 22 |
| HLA-C | C*07:02:01:03 | C*05:01:01:02 | NK Cell | 169736 | 10656 | 18 | 766606 | 74734 | 22 |
| 71 |  |  | B Cell | 156178 | 12134 | 16 | 574488 | 39984 | 18 |
| HLA-A | A*03:01:01e | - | CD4+ T Cell | 137752 | 5113 | 16 | 620110 | 38511 | 18 |
| HLA-B | **B*07:02:01** | B*57:03:01e | CD8+ T Cell | 146525 | 5451 | 16 | 678572 | 42659 | 18 |
| HLA-C | C*06:02:01:01 | C*07:18 | NK Cell | 165089 | 7795 | 16 | 763029 | 63050 | 18 |

| SAMPLE ID | Allele 1 | Allele 2 | Cell Type | Bw6 ABC Mean | | | w6/32 ABC Mean | | |
| --- | --- | --- | --- | --- | --- | --- | --- | --- | --- |
|  |  |  |  | Mean | SEM | N | Mean | SEM | N |
| 75 |  |  | B Cell | 240512 | 16553 | 7 | 992714 | 96627 | 9 |
| HLA-A | A*01:01:01:01 | A*02:01:01:01 | CD4+ T Cell | 161835 | 4133 | 7 | 705228 | 46996 | 9 |
| HLA-B | **B*15:01:01:01** | B*57:01:01 | CD8+ T Cell | 230069 | 10048 | 7 | 1040466 | 78646 | 9 |
| HLA-C | C*06:02:01:01 | C*03:04:01:01 | NK Cell | 203915 | 14495 | 7 | 1005940 | 101468 | 9 |
| 79 |  |  | B Cell | 185319 | 22198 | 6 | 579894 | 71099 | 8 |
| HLA-A | A*03:01:01:01 | A*31:01:02:01 | CD4+ T Cell | 149966 | 8642 | 6 | 546124 | 41998 | 8 |
| HLA-B | **B*40:01:02** | B*13:02:01 | CD8+ T Cell | 236555 | 22699 | 6 | 881740 | 63048 | 8 |
| HLA-C | C*06:02:01:01 | C*03:04:01:01 | NK Cell | 225387 | 10428 | 6 | 775747 | 75899 | 8 |
| 80 |  |  | B Cell | 230122 | 17380 | 12 | 776366 | 58091 | 12 |
| HLA-A | A*02:01:01:01 | - | CD4+ T Cell | 136466 | 7851 | 12 | 532995 | 29658 | 12 |
| HLA-B | **B*07:02:01** | B*44:02:01:01 | CD8+ T Cell | 165850 | 9626 | 12 | 661828 | 38676 | 12 |
| HLA-C | C*07:02:01:03 | C*05:01:01:02 | NK Cell | 199369 | 16789 | 12 | 778834 | 55700 | 12 |
| 91 |  |  | B Cell | 226006 | 23924 | 7 | 691633 | 94633 | 10 |
| HLA-A | A*02:01:01:01 | A*68:02:01:01 | CD4+ T Cell | 190373 | 8008 | 7 | 647687 | 58388 | 10 |
| HLA-B | **B*40:01:02** | B*44:02:01:01 | CD8+ T Cell | 227141 | 8540 | 7 | 806937 | 71203 | 10 |
| HLA-C | C*05:01:01:02 | C*03:03:01 | NK Cell | 199777 | 27003 | 7 | 711809 | 84772 | 10 |
| 94 |  |  | B Cell | 462436 | 18926 | 18 | 1230898 | 118265 | 23 |
| HLA-A | A*68:01:02:01 | A*01:01:01:01 | CD4+ T Cell | 237829 | 9934 | 18 | 714825 | 59652 | 23 |
| HLA-B | **B*08:01:01e1** | B*44:02:01:01 | CD8+ T Cell | 270848 | 10926 | 18 | 837990 | 69879 | 23 |
| HLA-C | C*05:01:01:02 | C*07:01:01:01 | NK Cell | 217485 | 15322 | 18 | 708490 | 82540 | 23 |
| 102 |  |  | B Cell | 256439 | 28782 | 4 | 622117 | 78944 | 6 |
| HLA-A | A*23:01:01 | A*25:01:01 | CD4+ T Cell | 262218 | 33585 | 4 | 758466 | 115539 | 6 |
| HLA-B | **B*08:01:01** | B*57:02:01 | CD8+ T Cell | 257775 | 28348 | 4 | 786115 | 106628 | 6 |
| HLA-C | C*07:01:01:01 | C*18:02e1 | NK Cell | 247195 | 41141 | 4 | 769356 | 113499 | 6 |
| 111 |  |  | B Cell | 179484 | 6801 | 13 | 565437 | 24081 | 15 |
| HLA-A | A*11:01:01:01 | A*03:01:01:01 | CD4+ T Cell | 154981 | 4623 | 13 | 580989 | 29471 | 15 |
| HLA-B | **B*35:01:01:02** | B*51:01:01:01 | CD8+ T Cell | 193827 | 5957 | 13 | 765280 | 35990 | 15 |
| HLA-C | C*01:02:01e1 | C*04:01:01:01 | NK Cell | 226745 | 11402 | 13 | 831608 | 40602 | 15 |
| 115 |  |  | B Cell | 338782 | 21544 | 6 | 1000422 | 58521 | 6 |
| HLA-A | A*01:01:01:01 | A*24:02:01:01 | CD4+ T Cell | 239918 | 16456 | 6 | 883100 | 69909 | 6 |
| HLA-B | **B*07:02:01** | B*57:01:01e1 | CD8+ T Cell | 250962 | 15686 | 6 | 895808 | 107802 | 6 |
| HLA-C | C*06:02:01:01 | C*07:02:01:03 | NK Cell | 271472 | 32054 | 6 | 947233 | 167654 | 6 |
| 120 |  |  | B Cell | 224987 | 30088 | 4 | 645255 | 80453 | 4 |
| HLA-A | A*32:01:01 | A*31:01:02:01 | CD4+ T Cell | 239446 | 15474 | 4 | 727674 | 34386 | 4 |
| HLA-B | **B*40:01:02e2** | B*44:02:01:01 | CD8+ T Cell | 251058 | 15214 | 4 | 774547 | 18520 | 4 |
| HLA-C | C*05:01:01:02 | C*03:04:01:01e | NK Cell | 217711 | 7635 | 4 | 614972 | 37433 | 4 |
| 121 |  |  | B Cell | 190823 | 10549 | 10 | 539091 | 26583 | 16 |
| HLA-A | A*01:01:01:01 | - | CD4+ T Cell | 195585 | 8482 | 10 | 533203 | 18089 | 16 |
| HLA-B | **B*08:01:01** | B*27:05:02e1 | CD8+ T Cell | 283403 | 11559 | 10 | 795882 | 26790 | 16 |
| HLA-C | C*02:07 | C*07:01:01:01 | NK Cell | 243315 | 11731 | 10 | 761274 | 28861 | 16 |

| SAMPLE ID | Allele 1 | Allele 2 | Cell Type | Bw6 ABC Mean | | | w6/32 ABC Mean | | |
| --- | --- | --- | --- | --- | --- | --- | --- | --- | --- |
|  |  |  |  | Mean | SEM | N | Mean | SEM | N |
| 124 |  |  | B Cell | 291826 | 9219 | 6 | 636843 | 34623 | 8 |
| HLA-A | A*32:01:01 | A*11:01:01:01 | CD4+ T Cell | 168684 | 4316 | 6 | 574260 | 41752 | 8 |
| HLA-B | **B*15:01:01:01** | B*53:01:01 | CD8+ T Cell | 226596 | 6434 | 6 | 765359 | 59260 | 8 |
| HLA-C | C*06:02:01:01 | C*04:01:01:01 | NK Cell | 215519 | 19285 | 6 | 676561 | 136242 | 8 |
| 128 |  |  | B Cell | 271198 | 26024 | 13 | 693573 | 33279 | 15 |
| HLA-A | A*11:01:01:01 | A*02:01:01:01 | CD4+ T Cell | 215687 | 17508 | 13 | 769691 | 51730 | 15 |
| HLA-B | **B*15:01:01:01** | B*44:02:01:01 | CD8+ T Cell | 252766 | 18313 | 13 | 915760 | 56466 | 15 |
| HLA-C | C*03:03:01e1 | C*05:01:01:02 | NK Cell | 213968 | 19810 | 13 | 695601 | 76937 | 15 |
| 130 |  |  | B Cell | 205725 | 7526 | 6 | 533317 | 25972 | 10 |
| HLA-A | A*01:01:01:01 | A*30:01:01 | CD4+ T Cell | 212414 | 3023 | 6 | 635477 | 25087 | 10 |
| HLA-B | **B*08:01:01** | B*13:02:01 | CD8+ T Cell | 350153 | 8219 | 6 | 1094903 | 45510 | 10 |
| HLA-C | C*07:01:01:01 | C*06:02:01:01 | NK Cell | 233131 | 3790 | 6 | 721555 | 31718 | 10 |
| 131 |  |  | B Cell | 278890 | 6700 | 6 | 537636 | 21736 | 8 |
| HLA-A | A*03:01:01:01 | A*24:02:01:05 | CD4+ T Cell | 158944 | 6600 | 6 | 438086 | 17719 | 8 |
| HLA-B | **B*18:01:01:02** | B*27:02:01 | CD8+ T Cell | 202186 | 8169 | 6 | 585334 | 24878 | 8 |
| HLA-C | C*12:03:01:01 | C*02:02:02:01 | NK Cell | 143338 | 14325 | 6 | 471964 | 27400 | 8 |
| 137 |  |  | B Cell | 275935 | 10479 | 16 | 718262 | 36784 | 20 |
| HLA-A | A*01:01:01:01 | - | CD4+ T Cell | 208370 | 7682 | 16 | 613849 | 39809 | 20 |
| HLA-B | **B*08:01:01** | B*37:01:01e1 | CD8+ T Cell | 313388 | 10726 | 16 | 934235 | 58158 | 20 |
| HLA-C | C*07:01:01:01 | C*06:02:01:01 | NK Cell | 209786 | 8787 | 16 | 620995 | 46623 | 20 |
| 141 |  |  | B Cell | 204538 | 7502 | 16 | 504233 | 23486 | 20 |
| HLA-A | A*02:01:01:01 | A*03:01:01:01 | CD4+ T Cell | 159140 | 4929 | 16 | 493942 | 21004 | 20 |
| HLA-B | **B*35:01:01:02** | B*44:02:01:01 | CD8+ T Cell | 226722 | 7636 | 16 | 746468 | 28958 | 20 |
| HLA-C | C*05:01:01:02 | C*04:01:01:01 | NK Cell | 179232 | 6612 | 16 | 636857 | 27331 | 20 |
| 166 |  |  | B Cell | 121773 | 3000 | 4 | 338018 | 19490 | 6 |
| HLA-A | A*01:01:01:01 | - | CD4+ T Cell | 190295 | 20435 | 4 | 540764 | 19594 | 6 |
| HLA-B | **B*08:01:01** | - | CD8+ T Cell | 227975 | 14167 | 4 | 650448 | 25079 | 6 |
| HLA-C | C*07:01:01:01 | - | NK Cell | 158861 | 7316 | 4 | 535809 | 32403 | 6 |
| 168 |  |  | B Cell | 103564 | 5616 | 4 | 370210 | 21599 | 9 |
| HLA-A | A*02:01:01:01 | A*11:01:01:01 | CD4+ T Cell | 127682 | 9798 | 4 | 509739 | 21106 | 9 |
| HLA-B | **B*35:01:01:02** | B*51:01:01:01 | CD8+ T Cell | 146428 | 10279 | 4 | 614469 | 24297 | 9 |
| HLA-C | C*15:02:01:01 | C*04:01:01:01 | NK Cell | 110822 | 9483 | 4 | 469479 | 20400 | 9 |
| 178 |  |  | B Cell | 214919 | 11735 | 14 | 555081 | 21833 | 18 |
| HLA-A | A*02:01:01:01 | A*01:01:01:01 | CD4+ T Cell | 230449 | 10949 | 14 | 639426 | 23368 | 18 |
| HLA-B | **B*08:01:01** | B*57:01:01 | CD8+ T Cell | 264377 | 12597 | 14 | 787512 | 28761 | 18 |
| HLA-C | C*06:02:01:01 | C*07:01:01:01 | NK Cell | 167842 | 7801 | 14 | 583752 | 28934 | 18 |
| 187 |  |  | B Cell | 231534 | 9163 | 10 | 530435 | 38884 | 10 |
| HLA-A | A*02:01:01:01 | A*01:01:01:01 | CD4+ T Cell | 138503 | 5308 | 10 | 408892 | 37302 | 10 |
| HLA-B | **B*35:01:01:02** | B*44:02:01:01 | CD8+ T Cell | 160824 | 7270 | 10 | 508472 | 49710 | 10 |
| HLA-C | C*05:01:01:02 | C*04:01:01:05 | NK Cell | 122063 | 8783 | 10 | 496038 | 52006 | 10 |

| SAMPLE ID | Allele 1 | Allele 2 | | | Cell Type | | Bw6 ABC Mean | | | | w6/32 ABC Mean | | | | |
| --- | --- | --- | --- | --- | --- | --- | --- | --- | --- | --- | --- | --- | --- | --- | --- |
|  |  |  |  |  |  |  | Mean | SEM | N | | Mean | | SEM | N | |
| 194 |  |  | | | B Cell | 142783 | | 11151 | | 10 | 428997 | | 25830 | 16 | |
| HLA-A | A*03:01:01:01 | A*01:01:01:01 | | | CD4+ T Cell | 129940 | | 9012 | | 10 | 534632 | | 31511 | 16 | |
| HLA-B | **B*07:02:01** | B*57:01:01 | | | CD8+ T Cell | 186059 | | 11876 | | 10 | 734497 | | 43474 | 16 | |
| HLA-C | C*07:02:01:03 | C*06:02:01:01 | | | NK Cell | 166060 | | 11377 | | 10 | 649102 | | 36294 | 16 | |
| 196 |  | |  | B Cell | | 154875 | | 9260 | 10 | | | 475801 | 45801 | | 12 |
| HLA-A | A*02:01:01:01 | | A*03:01:01:01 | CD4+ T Cell | | 171402 | | 7331 | 10 | | | 627135 | 43486 | | 12 |
| HLA-B | **B*07:02:01** | | B*44:02:01:01 | CD8+ T Cell | | 242252 | | 10312 | 10 | | | 849574 | 63209 | | 12 |
| HLA-C | C*05:01:01:02 | | C*07:02:01:03 | NK Cell | | 181957 | | 9322 | 10 | | | 669129 | 46911 | | 12 |
| 198 |  | |  | B Cell | | 209561 | | 6421 | 14 | | | 579358 | 12549 | | 18 |
| HLA-A | A*03:01:01:01 | | A*01:01:01:01 | CD4+ T Cell | | \176681 | | 4364 | 14 | | | 551187 | 16426 | | 18 |
| HLA-B | **B*08:01:01** | | B*57:01:01 | CD8+ T Cell | | 244492 | | 5100 | 14 | | | 789102 | 25929 | | 18 |
| HLA-C | C*06:02:01:01 | | C*07:01:01:01 | NK Cell | | 218712 | | 7746 | 14 | | | 757471 | 22645 | | 18 |
| 201 |  | |  | B Cell | | 249814 | | 8036 | 4 | | | 831029 | 61136 | | 6 |
| HLA-A | A*02:01:01:01 | | A*24:02:01:01 | CD4+ T Cell | | 203148 | | 5212 | 4 | | | 790921 | 49105 | | 6 |
| HLA-B | **B*07:02:01** | | - | CD8+ T Cell | | 274764 | | 8373 | 4 | | | 1027783 | 66292 | | 6 |
| HLA-C | C*07:02:01:03 | | - | NK Cell | | 301125 | | 41032 | 4 | | | 750435 | 87829 | | 6 |
| 206 |  | |  | B Cell | | 324419 | | 27954 | 4 | | | 863017 | 142342 | | 6 |
| HLA-A | - | | A*02:01:01:01 | CD4+ T Cell | | 153214 | | 6162 | 4 | | | 453107 | 33270 | | 6 |
| HLA-B | **B*18:01:01:02** | | B*44:02:01:01 | CD8+ T Cell | | 190544 | | 8406 | 4 | | | 532483 | 27363 | | 6 |
| HLA-C | C*07:01:01:01 | | C*05:01:01:02 | NK Cell | | 241902 | | 7943 | 4 | | | 778359 | 74959 | | 6 |
