# Supplementary material for: Variations in HLA-B cell surface expression, half-life and extracellular antigen receptivity
Source: eLife. 2018 Jul 10;7:e34961. doi: 10.7554/eLife.34961 (PMC6039183; doi:10.7554/eLife.34961)
Supplement: Figure 2—source data 1. — Calculated HLA-Bw6 half-lives on lymphocytes from donors with relevant HLA-B genotypes indicated. The complete HLA class I genotypes of the donors are specified in Figure 1—source data 1. Mean half-life values are shown along with standard errors of mean half-life values (SEM) and the number of measurements (N; from separate blood collections) used for calculating the mean values. [file elife-34961-fig2-data1.docx]

**Figure 2 - Source Data 1 HLA-Bw6 stability on lymphocytes**

Calculated HLA-Bw6 half-lives on lymphocytes from donors with relevant HLA-B genotypes indicated. The complete HLA class I genotypes of the donors are specified in Figure 1-Source Data 1. Mean half-life values are shown along with standard errors of mean half-life values (SEM) and the number of measurements (N; from separate blood collections) used for calculating the mean values.

| Donor ID: | HLA-B Allele | B Cells | | | NK Cells | | | CD4 T Cells | | | CD8 T Cells | | |
| --- | --- | --- | --- | --- | --- | --- | --- | --- | --- | --- | --- | --- | --- |
|  |  | Mean | SEM | N | Mean | SEM | N | Mean | SEM | N | Mean | SEM | N |
| 14 | B*07:02 | 16.05 | 1.66 | 3 | 12.83 | 2.96 | 3 | 11.98 | 0.89 | 3 | 13.59 | 1.87 | 3 |
| 31 | B*07:02 | 15.35 | 3.36 | 4 | 7.87 | 0.57 | 4 | 11.05 | 0.17 | 4 | 11.63 | 0.63 | 4 |
| 64 | B*07:02 | 27.69 | 5.86 | 5 | 16.84 | 7.03 | 5 | 18.45 | 4.62 | 5 | 17.93 | 4.31 | 5 |
| 71 | B*07:02 | 13.98 | 2.78 | 3 | 10.55 | 2.61 | 4 | 12.25 | 2.44 | 4 | 12.10 | 1.67 | 4 |
| 28 | B*08:01 | 20.09 | 2.21 | 4 | 25.37 | 9.27 | 4 | 16.53 | 1.30 | 4 | 27.13 | 4.10 | 4 |
| 55 | B*08:01 | 21.01 | 6.09 | 4 | 10.80 | 0.15 | 4 | 13.63 | 1.10 | 4 | 19.43 | 4.16 | 4 |
| 94 | B*08:01 | 37.43 | 9.48 | 5 | 14.86 | 2.46 | 6 | 26.61 | 4.89 | 6 | 22.97 | 4.98 | 6 |
| 121 | B*08:01 | 45.98 | 21.35 | 2 | 19.19 | 7.85 | 2 | 18.32 | 2.62 | 2 | 20.62 | 7.24 | 2 |
| 137 | B*08:01 | 20.06 | 1.23 | 7 | 15.31 | 2.38 | 5 | 18.68 | 1.88 | 7 | 20.39 | 5.44 | 6 |
| 178 | B*08:01 | 19.31 | 2.83 | 4 | 20.25 | 8.62 | 3 | 26.88 | 4.01 | 4 | 29.70 | 5.44 | 4 |
| 198 | B*08:01 | 14.17 | 2.97 | 3 | 11.17 | 1.56 | 2 | 16.58 | 5.89 | 2 | 16.55 | 6.30 | 2 |
| 8 | B*15:01 | 19.49 | 2.68 | 2 | 8.55 | 0.41 | 2 | 12.38 | 0.06 | 2 | 9.26 | 0.40 | 2 |
| 124 | B*15:01 | 19.40 | 1.89 | 2 | 11.51 | 1.82 | 2 | 16.79 | 0.04 | 2 | 15.29 | 0.54 | 2 |
| 128 | B*15:01 | 23.68 | 0.75 | 2 | 20.31 | 1.70 | 2 | 19.06 | 1.93 | 2 | 17.71 | 3.33 | 2 |
| 131 | B*18:01 | 17.85 | 0.86 | 2 | 7.76 | 0.19 | 2 | 12.64 | 2.11 | 2 | 11.14 | 1.24 | 2 |
| 206 | B*18:01 | 22.30 | 1.36 | 2 | 9.87 | 1.20 | 2 | 11.68 | 0.77 | 2 | 13.28 | 0.55 | 2 |
| 215 | B*18:01 | 24.49 | 6.64 | 2 | 7.66 | 0.59 | 2 | 15.59 | 0.48 | 2 | 15.46 | 1.45 | 2 |
| 24 | B*35:01 | 15.33 | 1.87 | 4 | 8.29 | 0.50 | 4 | 13.64 | 1.68 | 4 | 11.25 | 1.20 | 4 |
| 111 | B*35:01 | 16.64 | 0.64 | 2 | 9.32 | 1.97 | 2 | 10.81 | 1.15 | 2 | 10.83 | 0.35 | 2 |
| 141 | B*35:01 | 14.98 | 1.99 | 4 | 7.42 | 0.56 | 4 | 10.53 | 1.18 | 4 | 8.11 | 0.81 | 4 |
| 187 | B*35:01 | 13.52 | 1.99 | 4 | 5.77 | 0.24 | 4 | 9.56 | 0.52 | 4 | 8.41 | 0.29 | 4 |
| 91 | B*40:01 | 19.40 | 8.92 | 2 | 9.36 | 2.10 | 2 | 9.52 | 2.52 | 2 | 9.88 | 2.82 | 2 |
| 120 | B*40:01 | 16.31 | 1.39 | 3 | 17.28 | 7.12 | 3 | 14.32 | 3.31 | 3 | 13.71 | 2.90 | 3 |
