# Supplementary material for: Variations in HLA-B cell surface expression, half-life and extracellular antigen receptivity
Source: eLife. 2018 Jul 10;7:e34961. doi: 10.7554/eLife.34961 (PMC6039183; doi:10.7554/eLife.34961)
Supplement: Figure 3—source data 1. — Relevant HLA class I genotypes of donors and mean of ABC values measured with anti-Bw6 and W6/32 are shown for each lymphocyte or monocyte subset. The complete HLA class I genotypes of the donors are specified in Figure 1—source data 1. Standard errors of the mean (SEM) values and the number of replicate measurements (N; with separate blood collections) are indicated. [file elife-34961-fig3-data1.docx]

**Figure 3 - Source Data 1: T Cell and Monocyte Bw6 ABC Values**

Relevant HLA class I genotypes of donors and mean of ABC values measured with anti-Bw6 and W6/32 are shown for each lymphocyte or monocyte subset. The complete HLA class I genotypes of the donors are specified in Figure 1-Source Data 1. Standard errors of the mean (SEM) values and the number of replicate measurements (N; with separate blood collections) are indicated.

| Sample ID | Allele 1 | Cell Type | Bw6 ABC Mean | | | w6/32 ABC Mean | | |
| --- | --- | --- | --- | --- | --- | --- | --- | --- |
|  |  |  | Mean | SEM | N | Mean | SEM | N |
| 14 |  |  |  |  |  |  |  |  |
| HLA-B | B*07:02:01 | CD4+ T Cell | 118789 | 3807 | 4 | 373887 | 29822 | 4 |
|  |  | CD8+ T Cell | 191256 | 7333 | 4 | 573017 | 42349 | 4 |
|  |  | Monocytes | 392445 | 10493 | 4 | 1347993 | 88356 | 4 |
| 24 |  |  |  |  |  |  |  |  |
| HLA-B | B*35:01:01:02 | CD4+ T Cell | 114302 | 2527 | 4 | 339373 | 3467 | 4 |
|  |  | CD8+ T Cell | 149551 | 3235 | 4 | 445265 | 4112 | 4 |
|  |  | Monocytes | 306761 | 11566 | 4 | 999006 | 42493 | 4 |
| 31 |  |  |  |  |  |  |  |  |
| HLA-B | B*07:02:01 | CD4+ T Cell | 127222 | 3362 | 4 | 341357 | 10065 | 4 |
|  |  | CD8+ T Cell | 174344 | 1693 | 4 | 453282 | 9980 | 4 |
|  |  | Monocytes | 382345 | 10148 | 4 | 916761 | 39407 | 4 |
| 64 |  |  |  |  |  |  |  |  |
| HLA-B | B*07:02:01 | CD4+ T Cell | 142527 | 8009 | 4 | 395241 | 27797 | 4 |
|  |  | CD8+ T Cell | 177198 | 10499 | 4 | 481697 | 28420 | 4 |
|  |  | Monocytes | 458636 | 48204 | 4 | 1065103 | 63458 | 4 |
| 71 |  |  |  |  |  |  |  |  |
| HLA-B | B*07:02:01 | CD4+ T Cell | 134985 | 9632 | 4 | 469393 | 13757 | 4 |
|  |  | CD8+ T Cell | 146748 | 10721 | 4 | 511052 | 12885 | 4 |
|  |  | Monocytes | 333512 | 24225 | 4 | 1233233 | 19558 | 4 |
| 94 |  |  |  |  |  |  |  |  |
| HLA-B | B*08:01:01 | CD4+ T Cell | 190144 | 3777 | 4 | 446109 | 6858 | 4 |
|  |  | CD8+ T Cell | 219629 | 1707 | 4 | 533345 | 18226 | 4 |
|  |  | Monocytes | 299546 | 16251 | 4 | 1017014 | 65779 | 4 |
| 111 |  |  |  |  |  |  |  |  |
| HLA-B | B*35:01:01:02 | CD4+ T Cell | 165506 | 5153 | 4 | 518049 | 17212 | 4 |
|  |  | CD8+ T Cell | 213932 | 4920 | 4 | 690111 | 19525 | 4 |
|  |  | Monocytes | 520267 | 25569 | 4 | 1275868 | 49362 | 4 |
| 137 |  |  |  |  |  |  |  |  |
| HLA-B | B*08:01:01 | CD4+ T Cell | 190498 | 10115 | 4 | 421343 | 23380 | 4 |
|  |  | CD8+ T Cell | 288549 | 7214 | 4 | 650520 | 21196 | 4 |
|  |  | Monocytes | 227479 | 13135 | 4 | 824325 | 27091 | 4 |

| SAMPLE ID | Allele 1 | Cell Type | Bw6 ABC Mean | | | w6/32 ABC Mean | | |
| --- | --- | --- | --- | --- | --- | --- | --- | --- |
|  |  |  | Mean | SEM | N | Mean | SEM | N |
| 141 |  |  |  |  |  |  |  |  |
| HLA-B | B*35:01:01:02 | CD4+ T Cell | 147195 | 11547 | 4 | 416316 | 9760 | 4 |
|  |  | CD8+ T Cell | 210906 | 18188 | 4 | 646060 | 16554 | 4 |
|  |  | Monocytes | 392966 | 31422 | 4 | 1038441 | 43016 | 4 |
| 168 |  |  |  |  |  |  |  |  |
| HLA-B | B*35:01:01:02 | CD4+ T Cell | 127683 | 9798 | 4 | 464324 | 21516 | 4 |
|  |  | CD8+ T Cell | 146428 | 10280 | 4 | 564733 | 19729 | 4 |
|  |  | Monocytes | 410497 | 66150 | 4 | 1148943 | 103012 | 4 |
| 178 |  |  |  |  |  |  |  |  |
| HLA-B | B*08:01:01 | CD4+ T Cell | 229801 | 12677 | 6 | 596395 | 48451 | 6 |
|  |  | CD8+ T Cell | 269894 | 16362 | 6 | 731221 | 58997 | 6 |
|  |  | Monocytes | 216306 | 18188 | 6 | 1156690 | 67921 | 6 |
| 187 |  |  |  |  |  |  |  |  |
| HLA-B | B*35:01:01:02 | CD4+ T Cell | 128062 | 6604 | 4 | 317645 | 13331 | 4 |
|  |  | CD8+ T Cell | 143512 | 4777 | 4 | 393057 | 14281 | 4 |
|  |  | Monocytes | 312398 | 7574 | 4 | 667981 | 24147 | 4 |
| 198 |  |  |  |  |  |  |  |  |
| HLA-B | B*08:01:01 | CD4+ T Cell | 170897 | 1473 | 4 | 459097 | 25240 | 4 |
|  |  | CD8+ T Cell | 257132 | 2748 | 4 | 694959 | 39623 | 4 |
|  |  | Monocytes | 308704 | 8056 | 4 | 1109658 | 57942 | 4 |
