# Supplementary material for: Variations in HLA-B cell surface expression, half-life and extracellular antigen receptivity
Source: eLife. 2018 Jul 10;7:e34961. doi: 10.7554/eLife.34961 (PMC6039183; doi:10.7554/eLife.34961)
Supplement: Figure 3—source data 2. — Calculated HLA-Bw6 half-lives on leukocytes from donors with relevant HLA-B genotypes indicated. The complete HLA class I genotypes of the donors are specified in Figure 1—source data 1. Mean half-life values are shown along with standard errors of mean half-life values (SEM) and the number of measurements (N; from separate blood collections) used for calculating the mean values. [file elife-34961-fig3-data2.docx]

**Figure 3 - Source Data 2 HLA-Bw6 stability on monocyte, CD4^+^ T cell and CD8^+^ T cell**

Calculated HLA-Bw6 half-lives on leukocytes from donors with relevant HLA-B genotypes indicated. The complete HLA class I genotypes of the donors are specified in Figure 1-Source Data 1. Mean half-life values are shown along with standard errors of mean half-life values (SEM) and the number of measurements (N; from separate blood collections) used for calculating the mean values.

| Donor ID: | HLA-B Allele | Monocytes | | | CD4 T Cells | | | CD8 T Cells | | |
| --- | --- | --- | --- | --- | --- | --- | --- | --- | --- | --- |
|  |  | Mean | SEM | N | Mean | SEM | N | Mean | SEM | N |
| 14 | B*07:02 | 18.49 |  | 1 | 10.40 |  | 1 | 11.32 |  | 1 |
| 31 | B*07:02 | 8.88 |  | 1 | 10.83 | 0.13 | 2 | 10.75 | 0.90 | 2 |
| 64 | B*07:02 | 10.63 | 1.36 | 2 | 24.46 | 12.23 | 2 | 24.30 | 10.42 | 2 |
| 71 | B*07:02 | 5.68 | 0.56 | 2 | 13.33 | 5.42 | 2 | 12.73 | 3.98 | 2 |
| 28 | B*08:01 | 5.06 |  | 1 | 19.40 |  | 1 | 26.20 |  | 1 |
| 94 | B*08:01 | 13.56 |  | 1 | 17.47 | 7.43 | 2 | 18.20 | 8.85 | 2 |
| 137 | B*08:01 | 7.24 | 0.90 | 2 | 21.32 | 2.63 | 2 | 23.78 |  | 1 |
| 178 | B*08:01 | 7.74 | 1.39 | 2 | 26.02 | 4.18 | 2 | 37.68 | 7.08 | 2 |
| 24 | B*35:01 | 8.19 | 1.63 | 2 | 13.48 | 3.93 | 2 | 9.40 | 0.26 | 2 |
| 141 | B*35:01 | 10.69 | 1.05 | 2 | 12.50 | 0.02 | 2 | 9.41 | 0.07 | 2 |
| 187 | B*35:01 | 9.37 | 1.20 | 2 | 10.12 | 0.88 | 2 | 8.68 | 0.52 | 2 |
