# Supplementary material for: Variations in HLA-B cell surface expression, half-life and extracellular antigen receptivity
Source: eLife. 2018 Jul 10;7:e34961. doi: 10.7554/eLife.34961 (PMC6039183; doi:10.7554/eLife.34961)
Supplement: Figure 4—source data 1. — The data represents one imaging cytometry experiment performed on two donors: 94 and 64. Genotypes for donors 94 and 64 are indicated in Figure 1—source data 1. In monocytes and CD4+ T cells, Bw6 colocalization is quantified with three different intracellular markers: AP-1 (top), calreticulin (middle), and LAMP-1 (bottom). In imaging cytometry experiments, co-localization is quantified as Bright Detail Similarity (BDS), which is the degree of overlap between the two markers of interest. The red columns represent cell population gates with a high degree of co-localization, yellow columns represent cells with intermediate co-localization, and blue columns represent cells with low co-localization. Intermediate co-localization was calculated only for Bw6/AP-1 co-localization. The first row for each donor is the quantification of the cell count within each gate, the second row is the percentage of cells within a gate, relative to the total number of cells in the previous gate, and the final row is the median BDS for each population. In each cell population, the Bw6+ M2+ column represents cells that are double positive for Bw6 and the second co-localization marker (Marker 2; M2). M2 is AP-1 for the top table, calreticulin for the middle table, and LAMP-1 for the bottom table. [file elife-34961-fig4-data1.docx]

**Figure 4 – Source Data 1: Imaging cytometry co-localization source data**

The data represents one imaging cytometry experiment performed on two donors: 94 and 64. Genotypes for donors 94 and 64 are indicated in Figure 1-Source Data 1. In monocytes and CD4^+^ T cells, Bw6 colocalization is quantified with three different intracellular markers: AP-1 (top), calreticulin (middle), and LAMP-1 (bottom). In imaging cytometry experiments, co-localization is quantified as Bright Detail Similarity (BDS), which is the degree of overlap between the two markers of interest. The red columns represent cell population gates with a high degree of co-localization, yellow columns represent cells with intermediate co-localization, and blue columns represent cells with low co-localization. Intermediate co-localization was calculated only for Bw6/AP-1 co-localization. The first row for each donor is the quantification of the cell count within each gate, the second row is the percentage of cells within a gate, relative to the total number of cells in the previous gate, and the final row is the median BDS for each population. In each cell population, the Bw6^+^ M2^+^ column represents cells that are double positive for Bw6 and the second co-localization marker (Marker 2; M2). M2 is AP-1 for the top table, calreticulin for the middle table, and LAMP-1 for the bottom table.

| Population | | | | | | **CD4^+^ T cell co-localization** | | | | | **Monocyte co-localization** | | | | |
| --- | --- | --- | --- | --- | --- | --- | --- | --- | --- | --- | --- | --- | --- | --- | --- |
|  |  |  |  |  |  | CD3^+^ CD8^-^ | Bw6^+^ M2^+^ | High | Int. | Low | CD3^-^ CD14^+^ | Bw6^+^ M2^+^ | High | Int. | Low |
| **Bw6/AP-1 co-localization** | **Donor 94** | | | | Count | 11005 | 10955 | 2852 | 6243 | 673 | 1729 | 1701 | 335 | 1003 | 181 |
|  | %Gated | | | | | 35.48 | 99.55 | 26.03 | 56.99 | 6.14 | 5.57 | 98.38 | 19.69 | 58.97 | 10.64 |
|  | Bright Detail Similarity R3_MC_Ch02_Ch04, Median | | | | | 1.93 | 1.93 | 2.49 | 1.80 | 1.03 | 1.84 | 1.84 | 2.48 | 1.77 | 0.91 |
|  | **Donor 64** | | | | Count | 6259 | 6169 | 1892 | 3391 | 323 | 859 | 848 | 157 | 410 | 173 |
|  | %Gated | | | | | 21.27 | 98.56 | 30.67 | 54.97 | 5.24 | 2.92 | 98.72 | 18.51 | 48.35 | 20.40 |
|  | Bright Detail Similarity R3_MC_Ch02_Ch04, Median | | | | | 1.97 | 1.97 | 2.52 | 1.77 | 1.02 | 1.80 | 1.80 | 2.49 | 1.82 | 1.19 |
| **Bw6/calreticulin co-localization** | **Donor 94** | Count | | | | 15240 | 15215 | 245 |  | 4989 | 2698 | 2683 | 8 |  | 1900 |
|  | %Gated | | | | | 38.41 | 99.84 | 1.61 |  | 32.79 | 6.8 | 99.44 | 0.3 |  | 70.82 |
|  | Bright Detail Similarity R3_MC_Ch02_Ch04, Median | | | | | 1.33 | 1.33 | 2.377 |  | 0.9883 | 1.236 | 1.235 | 2.374 |  | 1.105 |
|  | **Donor 64** | | Count | | | 7288 | 7229 | 189 |  | 2533 | 1126 | 1102 | 13 |  | 718 |
|  | %Gated | | | | | 23.18 | 99.19 | 2.61 |  | 35.04 | 3.58 | 97.87 | 1.18 |  | 65.15 |
|  | Bright Detail Similarity R3_MC_Ch02_Ch04, Median | | | | | 1.318 | 1.317 | 2.43 |  | 0.9636 | 1.281 | 1.279 | 2.4 |  | 1.121 |
| **Bw6/LAMP-1 co-localization** | **Donor 94** | | | Count | | 12934 | 12876 | 260 |  | 8492 | 2560 | 2546 | 10 |  | 2374 |
|  | %Gated | | | | | 37.33 | 99.55 | 2.02 |  | 65.95 | 7.39 | 99.45 | 0.39 |  | 93.24 |
|  | Bright Detail Similarity R3_MC_Ch02_Ch04, Median | | | | | 1.083 | 1.083 | 2.47 |  | 0.9055 | 0.9279 | 0.9271 | 2.484 |  | 0.904 |
|  | **Donor 64** | | Count | | | 8690 | 8682 | 341 |  | 5482 | 1454 | 1445 | 15 |  | 1241 |
|  | %Gated | | | | | 26.26 | 99.91 | 3.93 |  | 63.14 | 4.39 | 99.38 | 1.04 |  | 85.88 |
|  | Bright Detail Similarity R3_MC_Ch02_Ch04, Median | | | | | 1.103 | 1.103 | 2.461 |  | 0.8752 | 1.035 | 1.035 | 2.523 |  | 0.9623 |
