# Supplementary material for: Variations in HLA-B cell surface expression, half-life and extracellular antigen receptivity
Source: eLife. 2018 Jul 10;7:e34961. doi: 10.7554/eLife.34961 (PMC6039183; doi:10.7554/eLife.34961)
Supplement: Figure 5—source data 1. — Peptide receptivity (HC10 ratios (binding/control peptide)) in lymphocytes and monocytes. Full donor genotypes are indicated in Figure 1—source data 1. [file elife-34961-fig5-data1.docx]

**Figure 5 - Source Data 1: PBMC peptide receptivity source data**

Peptide receptivity (HC10 ratios (binding/control peptide)) in lymphocytes and monocytes. Full donor genotypes are indicated in Figure 1, Source Data 1.

| Donor ID | Allele | Binding/Control | CD4 T Cells | | CD8 T Cells | | B Cells | | NK Cells | | Monocytes | |
| --- | --- | --- | --- | --- | --- | --- | --- | --- | --- | --- | --- | --- |
|  |  |  | mean | SEM | mean | SEM | mean | SEM | mean | SEM | mean | SEM |
| 28 | B*08:01 | HSK / HSD | 1.00 | 0.03 | 0.95 | 0.03 | 0.99 | 0.03 | 0.98 | 0.04 | 0.95 | 0.02 |
|  |  | GPK / GPD | 0.98 | 0.02 | 0.98 | 0.05 | 0.98 | 0.03 | 1.00 | 0.01 | 1.01 | 0.04 |
|  |  | QIK / QID | 1.06 | 0.06 | 1.01 | 0.04 | 1.05 | 0.03 | 1.04 | 0.01 | 1.07 | 0.04 |
| 94 | B*08:01 | HSK / HSD | 0.75 | 0.13 | 0.77 | 0.16 | 0.94 | 0.01 | 0.73 | 0.17 | 1.01 | 0.03 |
|  |  | GPK / GPD | 1.02 | 0.02 | 0.99 | 0.03 | 1.00 | 0.00 | 1.01 | 0.16 | 0.99 | 0.05 |
|  |  | QIK / QID | 0.89 | 0.01 | 0.92 | 0.03 | 1.02 | 0.02 | 1.01 | 0.02 | 0.91 | 0.01 |
| 137 | B*08:01 | HSK / HSD | 1.29 | 0.46 | 1.31 | 0.53 | 1.09 | 0.06 | 1.23 | 0.28 | 1.28 | 0.26 |
|  |  | GPK / GPD | 1.21 | 0.07 | 1.34 | 0.10 | 1.04 | 0.02 | 1.14 | 0.06 | 1.16 | 0.06 |
|  |  | QIK / QID | 1.14 | 0.12 | 0.98 | 0.20 | 1.01 | 0.04 | 1.12 | 0.09 | 1.01 | 0.08 |
| 137 | B*08:01 | HSK / HSD | 1.00 | 0.04 | 1.15 | 0.06 | 1.00 | 0.04 | 1.11 | 0.06 | 1.16 | 0.02 |
|  |  | GPK / GPD | 1.01 | 0.01 | 1.03 | 0.06 | 0.99 | 0.05 | 1.02 | 0.00 | 1.06 | 0.13 |
|  |  | QIK / QID | 1.12 | 0.10 | 1.09 | 0.10 | 1.04 | 0.05 | 1.14 | 0.08 | 1.06 | 0.05 |
| 178 | B*08:01 | HSK / HSD | 0.96 | 0.01 | 1.03 | 0.01 | 0.98 | 0.00 | 1.00 | 0.00 | 1.12 | 0.00 |
|  |  | GPK / GPD | 0.99 |  | 1.09 |  | 1.00 |  | 1.04 |  | 1.14 |  |
|  |  | QIK / QID | 1.03 | 0.01 | 0.98 | 0.04 | 1.02 | 0.03 | 1.00 | 0.03 | 1.02 | 0.02 |
| 178 | B*08:01 | HSK / HSD | 1.11 | 0.02 | 0.91 | 0.05 | 1.13 | 0.02 | 0.98 | 0.05 | 0.80 | 0.06 |
|  |  | GPK / GPD | 1.04 | 0.05 | 0.94 | 0.02 | 1.00 | 0.05 | 0.99 | 0.07 | 0.91 | 0.02 |
|  |  | QIK / QID | 0.90 | 0.01 | 1.05 | 0.09 | 1.01 | 0.03 | 1.08 | 0.09 | 1.08 | 0.11 |
| 198 | B*08:01 | HSK / HSD | 0.99 | 0.02 | 1.14 | 0.04 | 0.97 | 0.01 | 1.04 | 0.02 | 1.12 | 0.01 |
|  |  | GPK / GPD | 1.03 | 0.09 | 1.01 | 0.22 | 1.01 | 0.06 | 1.10 | 0.07 | 1.20 | 0.07 |
|  |  | QIK / QID | 1.06 | 0.04 | 0.98 | 0.18 | 1.03 | 0.02 | 1.07 | 0.03 | 1.01 | 0.10 |
| 24 | B*35:01 | HPV / HGV | 0.73 | 0.17 | 0.89 | 0.26 | 0.60 | 0.01 | 0.84 | 0.12 | 1.08 | 0.15 |
|  |  | EPL / EGL | 0.86 | 0.11 | 1.21 | 0.12 | 0.67 | 0.02 | 0.88 | 0.05 | 1.17 | 0.04 |
|  |  | HPN / HGN | 1.04 | 0.04 | 1.14 | 0.06 | 0.92 | 0.01 | 1.01 | 0.02 | 1.05 | 0.04 |
| 24 | B*35:01 | HPV / HGV | 0.82 | 0.02 | 0.78 | 0.04 | 0.83 | 0.13 | 0.88 | 0.02 | 0.89 | 0.05 |
|  |  | EPL / EGL | 0.91 | 0.02 | 1.02 | 0.08 | 1.12 | 0.12 | 0.95 | 0.01 | 1.05 | 0.04 |
|  |  | HPN / HGN | 1.02 | 0.02 | 1.08 | 0.15 | 0.99 | 0.04 | 1.01 | 0.01 | 1.00 | 0.06 |
| 141 | B*35:01 | HPV / HGV | 0.76 | 0.02 | 0.92 | 0.01 | 0.72 | 0.01 | 0.85 | 0.03 | 1.01 | 0.01 |
|  |  | EPL / EGL | 0.70 | 0.01 | 0.91 | 0.01 | 0.71 | 0.03 | 0.83 | 0.01 | 1.14 | 0.01 |
|  |  | HPN / HGN | 0.97 | 0.01 | 1.00 | 0.03 | 0.95 | 0.01 | 1.01 | 0.00 | 1.01 | 0.03 |
| 187 | B*35:01 | HPV / HGV | 0.73 | 0.04 | 0.99 | 0.06 | 0.62 | 0.00 | 0.84 | 0.04 | 1.05 | 0.06 |
|  |  | EPL / EGL | 0.46 | 0.09 | 0.80 | 0.20 | 0.65 | 0.02 | 0.67 | 0.14 | 1.01 | 0.15 |
|  |  | HPN / HGN | 0.59 | 0.06 | 0.60 | 0.17 | 0.89 | 0.01 | 0.72 | 0.08 | 0.76 | 0.05 |

| Donor ID | Allele | Binding/Control | CD4 T Cells | | CD8 T Cells | | B Cells | | NK Cells | | Monocytes | |
| --- | --- | --- | --- | --- | --- | --- | --- | --- | --- | --- | --- | --- |
|  |  |  | mean | SEM | mean | SEM | mean | SEM | mean | SEM | mean | SEM |
| 187 | B*35:01 | HPV / HGV | 0.88 | 0.01 | 0.91 | 0.02 | 0.90 | 0.09 | 0.93 | 0.01 | 0.96 | 0.04 |
|  |  | EPL / EGL | 0.86 | 0.00 | 0.85 | 0.01 | 0.81 | 0.10 | 0.91 | 0.01 | 0.97 | 0.03 |
|  |  | HPN / HGN | 1.02 | 0.02 | 1.03 | 0.10 | 0.97 | 0.03 | 1.02 | 0.02 | 1.07 | 0.13 |
| 71 | B*07:02 | RPPI / RKPI | 0.67 | 0.02 | 0.95 | 0.04 | 0.76 | 0.01 | 0.89 | 0.02 | 0.97 | 0.03 |
|  |  | QPRA / QKRA | 0.94 | 0.01 | 1.04 | 0.01 | 0.92 | 0.02 | 1.04 | 0.02 | 1.06 | 0.02 |
|  |  | TPRV / TKRV | 0.80 | 0.01 | 0.99 | 0.03 | 0.84 | 0.01 | 0.93 | 0.02 | 0.98 | 0.02 |
| 64 | B*07:02 | RPPI / RKPI | 0.73 | 0.03 | 0.95 | 0.05 | 0.81 | 0.05 | 0.91 | 0.05 | 0.99 | 0.01 |
|  |  | QPRA / QKRA | 0.82 | 0.05 | 1.02 | 0.08 | 0.83 | 0.03 | 0.99 | 0.04 | 1.04 | 0.04 |
|  |  | TPRV / TKRV | 0.65 | 0.03 | 0.86 | 0.02 | 0.80 | 0.01 | 0.90 | 0.01 | 0.96 | 0.02 |
| 31 | B*07:02 | RPPI / RKPI | 0.95 | 0.14 | 1.01 | 0.09 | 0.73 | 0.05 | 1.06 | 0.16 | 0.99 | 0.02 |
|  |  | QPRA / QKRA | 0.87 | 0.01 | 0.93 | 0.01 | 0.85 | 0.03 | 0.96 | 0.01 | 1.03 | 0.02 |
|  |  | TPRV / TKRV | 0.88 | 0.04 | 0.92 | 0.03 | 0.80 | 0.02 | 0.92 | 0.04 | 0.95 | 0.01 |
| 14 | B*07:02 | RPPI / RKPI | 0.68 | 0.06 | 0.79 | 0.03 | 0.68 | 0.01 | 0.87 | 0.01 | 0.95 | 0.02 |
|  |  | QPRA / QKRA | 0.92 | 0.02 | 0.97 | 0.03 | 0.85 | 0.02 | 1.04 | 0.02 | 1.02 | 0.02 |
|  |  | TPRV / TKRV | 0.71 | 0.09 | 0.86 | 0.09 | 0.75 | 0.01 | 0.93 | 0.09 | 0.92 | 0.01 |
| 14 | B*07:02 | RPPI / RKPI | 0.86 | 0.02 | 0.87 | 0.04 | 0.94 | 0.12 | 0.94 | 0.03 | 0.92 | 0.03 |
|  |  | QPRA / QKRA | 0.92 | 0.01 | 0.96 | 0.02 | 0.93 | 0.10 | 0.99 | 0.01 | 0.98 | 0.03 |
|  |  | TPRV / TKRV | 0.93 | 0.01 | 0.97 | 0.04 | 0.94 | 0.06 | 1.02 | 0.02 | 1.01 | 0.03 |
| 31 | B*07:02 | RPPI / RKPI | 0.90 | 0.02 | 0.97 | 0.05 | 0.77 | 0.09 | 0.99 | 0.01 | 1.04 | 0.03 |
|  |  | QPRA / QKRA | 0.91 | 0.03 | 1.16 | 0.04 | 0.87 | 0.07 | 1.01 | 0.02 | 1.06 | 0.00 |
|  |  | TPRV / TKRV | 0.93 | 0.02 | 1.02 | 0.02 | 0.95 | 0.17 | 1.01 | 0.01 | 1.07 | 0.03 |
| 64 | B*07:02 | RPPI / RKPI | 0.90 | 0.01 | 0.88 | 0.02 | 0.72 | 0.06 | 0.90 | 0.02 | 0.95 | 0.03 |
|  |  | QPRA / QKRA | 0.98 | 0.02 | 0.94 | 0.06 | 0.72 | 0.03 | 0.99 | 0.03 | 0.95 | 0.06 |
|  |  | TPRV / TKRV | 0.92 | 0.01 | 0.91 | 0.03 | 0.82 | 0.03 | 0.99 | 0.02 | 1.07 | 0.02 |
