# Supplementary material for: Variations in HLA-B cell surface expression, half-life and extracellular antigen receptivity
Source: eLife. 2018 Jul 10;7:e34961. doi: 10.7554/eLife.34961 (PMC6039183; doi:10.7554/eLife.34961)
Supplement: Figure 6—source data 1. — HLA class I genotypes of donors used for Bw4 measurements, and mean of ABC values measured with anti-Bw4 and W6/32 are shown for each lymphocyte subset. The HLA-B-Bw4 allele of each donor is highlighted in bold. Standard errors of the mean (SEM) values and the number of replicate measurements (N; with separate blood collections) are indicated. [file elife-34961-fig6-data1.docx]

**Figure 6 – Source Data 1: Bw4 ABC Values.**

HLA class I genotypes of donors used for Bw4 measurements, and mean of ABC values measured with anti-Bw4 and W6/32 are shown for each lymphocyte subset. The HLA-B-Bw4 allele of each donor is highlighted in bold. Standard errors of the mean (SEM) values and the number of replicate measurements (N; with separate blood collections) are indicated.

| Donor ID | Allele 1 | Allele 2 | Cell Type | Bw4 ABC Mean | | | w6/32 ABC Mean | | |
| --- | --- | --- | --- | --- | --- | --- | --- | --- | --- |
|  |  |  |  | Mean | SEM | N | Mean | SEM | N |
| 5 |  |  | B Cell | 27790 | 3771 | 7 | 581013 | 69160 | 9 |
| HLA-A | A*02:01:01:01 | A*34:02:01 | CD4+ T Cell | 8233 | 1912 | 7 | 571605 | 49465 | 9 |
| HLA-B | B*35:01:01:02 | **B*44:02:01:01** | CD8+ T Cell | 8117 | 1927 | 7 | 679561 | 55661 | 9 |
| HLA-C | C*05:01:01:02 | C*16:01:01 | NK Cell | 9156 | 2187 | 7 | 651834 | 82201 | 9 |
| 8 |  |  | B Cell | 36731 | 2136 | 9 | 584299 | 48069 | 10 |
| HLA-A | A*01:01:01:01 | A*03:01:01:01 | CD4+ T Cell | 9388 | 911 | 9 | 678588 | 53908 | 10 |
| HLA-B | B*15:01:01 | **B*37:01:01** | CD8+ T Cell | 10706 | 993 | 9 | 836030 | 57813 | 10 |
| HLA-C | C*01:02:01 | C*06:02:01:01 | NK Cell | 12016 | 828 | 9 | 706679 | 58151 | 10 |
| 12 |  |  | B Cell | 46482 | 4855 | 3 | 761031 | 77301 | 9 |
| HLA-A | A*03:01:01:01 | A*01:01:01:01 | CD4+ T Cell | 7739 | 355 | 3 | 547346 | 40167 | 9 |
| HLA-B | B*08:01:01 | **B*44:02:01:01** | CD8+ T Cell | 6922 | 783 | 3 | 577277 | 47404 | 9 |
| HLA-C | C*05:01:01:02 | C*07:01:01:01 | NK Cell | 12130 | 1028 | 3 | 612580 | 59967 | 9 |
| 14 |  |  | B Cell | 49158 | 3520 | 13 | 611082 | 48036 | 21 |
| HLA-A | A*02:01:01:01 | A*03:01:01:01 | CD4+ T Cell | 9361 | 898 | 13 | 524415 | 31582 | 21 |
| HLA-B | B*07:02:01 | **B*51:01:01:01** | CD8+ T Cell | 12973 | 940 | 13 | 764337 | 44657 | 21 |
| HLA-C | C*15:02:01:01 | C*07:02:01:03 | NK Cell | 14082 | 756 | 13 | 599188 | 41974 | 21 |
| 20 |  |  | B Cell | 57021 | 6846 | 12 | 863865 | 75918 | 12 |
| HLA-A | A*02:01:01:01 | - | CD4+ T Cell | 13188 | 3493 | 12 | 761171 | 66406 | 12 |
| HLA-B | B*07:02:01 | **B*37:01:01** | CD8+ T Cell | 20730 | 4673 | 12 | 1247612 | 104350 | 12 |
| HLA-C | C*07:02:01:03 | C*06:02:01:01 | NK Cell | 22754 | 5021 | 12 | 759299 | 122420 | 12 |
| 23 |  |  | B Cell | 97153 | 12275 | 2 | 1295804 | 72881 | 2 |
| HLA-A | A*01:01:01:01 | - | CD4+ T Cell | 17579 | 161 | 2 | 808929 | 69756 | 2 |
| HLA-B | B*40:06:01:02 | **B*57:01:01** | CD8+ T Cell | 20231 | 103 | 2 | 1096845 | 71839 | 2 |
| HLA-C | C*06:02:01:01 | C*15:02:01:01 | NK Cell | 35186 | 4314 | 2 | 1324831 | 72742 | 2 |
| 28 |  |  | B Cell | 22235 | 1502 | 10 | 520365 | 44202 | 14 |
| HLA-A | A*02:01:01:01 | A*01:01:01:01 | CD4+ T Cell | 5963 | 1190 | 10 | 582195 | 51046 | 14 |
| HLA-B | B*08:01:01 | **B*51:01:01:01** | CD8+ T Cell | 6755 | 1250 | 10 | 660475 | 59808 | 14 |
| HLA-C | C*15:13 | C*07:01:01:01 | NK Cell | 6770 | 1119 | 10 | 688130 | 74454 | 14 |
| 62 |  |  | B Cell | 31599 | 1797 | 8 | 522232 | 45945 | 8 |
| HLA-A | A*01:01:01:01 | A*02:01:01:01 | CD4+ T Cell | 6661 | 1508 | 8 | 582129 | 46267 | 8 |
| HLA-B | B*08:01:01 | **B*51:01:01:01** | CD8+ T Cell | 12192 | 1381 | 8 | 897742 | 70777 | 8 |
| HLA-C | C*07:01:01:01 | C*14:02:01 | NK Cell | 14076 | 1468 | 8 | 713778 | 82664 | 8 |

| SAMPLE ID | | Allele 1 | | Allele 2 | Cell Type | Bw4 ABC Mean | | | w6/32 ABC Mean | | |
| --- | --- | --- | --- | --- | --- | --- | --- | --- | --- | --- | --- |
|  | | | | | | Mean | SEM | N | Mean | SEM | N |
| 64 |  | |  | | B Cell | 28086 | 1774 | 16 | 69768 | 61970 | 22 |
| HLA-A | A*03:01:01:01 | | A*02:01:01:01 | | CD4+ T Cell | 8297 | 458 | 16 | 607467 | 57827 | 22 |
| HLA-B | B*07:02:01 | | **B*44:02:01:01** | | CD8+ T Cell | 8354 | 445 | 16 | 699273 | 68952 | 22 |
| HLA-C | C*07:02:01:03 | | C*05:01:01:02 | | NK Cell | 11747 | 957 | 16 | 766606 | 74734 | 22 |
| 75 |  | |  | | B Cell | 75369 | 9494 | 7 | 992714 | 96627 | 9 |
| HLA-A | A*01:01:01:01 | | A*02:01:01:01 | | CD4+ T Cell | 14437 | 1141 | 7 | 705228 | 46996 | 9 |
| HLA-B | B*15:01:01:01 | | **B*57:01:01** | | CD8+ T Cell | 16878 | 1673 | 7 | 1040466 | 78646 | 9 |
| HLA-C | C*06:02:01:01 | | C*03:04:01:01 | | NK Cell | 20721 | 2325 | 7 | 1005940 | 101468 | 9 |
| 79 |  | |  | | B Cell | 34108 | 3662 | 6 | 579894 | 71099 | 8 |
| HLA-A | A*03:01:01:01 | | A*31:01:02:01 | | CD4+ T Cell | 8308 | 898 | 6 | 546124 | 41998 | 8 |
| HLA-B | B*40:01:02 | | **B*13:02:01** | | CD8+ T Cell | 12307 | 1200 | 6 | 881740 | 63048 | 8 |
| HLA-C | C*06:02:01:01 | | C*03:04:01:01 | | NK Cell | 12422 | 439 | 6 | 775747 | 75899 | 8 |
| 80 |  | |  | | B Cell | 45062 | 4170 | 8 | 776366 | 58091 | 12 |
| HLA-A | A*02:01:01:01 | | - | | CD4+ T Cell | 7999 | 889 | 8 | 532995 | 29658 | 12 |
| HLA-B | B*07:02:01 | | **B*44:02:01:01** | | CD8+ T Cell | 8353 | 1048 | 8 | 661828 | 38676 | 12 |
| HLA-C | C*07:02:01:03 | | C*05:01:01:02 | | NK Cell | 15859 | 1592 | 8 | 778834 | 55700 | 12 |
| 91 |  | |  | | B Cell | 32673 | 3332 | 8 | 691633 | 94633 | 10 |
| HLA-A | A*02:01:01:01 | | A*68:02:01:01 | | CD4+ T Cell | 10426 | 1534 | 8 | 647687 | 58388 | 10 |
| HLA-B | B*40:01:02 | | **B*44:02:01:01** | | CD8+ T Cell | 10559 | 1762 | 8 | 806937 | 71203 | 10 |
| HLA-C | C*05:01:01:02 | | C*03:03:01 | | NK Cell | 14422 | 1983 | 8 | 711809 | 84772 | 10 |
| 94 |  | |  | | B Cell | 47349 | 4939 | 10 | 1230898 | 118265 | 23 |
| HLA-A | A*68:01:02:01 | | A*01:01:01:01 | | CD4+ T Cell | 7524 | 949 | 10 | 714825 | 59652 | 23 |
| HLA-B | B*08:01:01e1 | | **B*44:02:01:01** | | CD8+ T Cell | 8344 | 957 | 10 | 837990 | 69879 | 23 |
| HLA-C | C*05:01:01:02 | | C*07:01:01:01 | | NK Cell | 8839 | 1371 | 10 | 708490 | 82540 | 23 |
| 106 |  | |  | | B Cell | 47042 | 5658 | 4 | 727042 | 3987 | 4 |
| HLA-A | A*02:01:01:01 | | A*26:01:01:01 | | CD4+ T Cell | 9269 | 322 | 4 | 692906 | 10451 | 4 |
| HLA-B | B*40:01:02 | | **B*27:05:02** | | CD8+ T Cell | 16012 | 442 | 4 | 923928 | 9221 | 4 |
| HLA-C | C*03:04:01:01 | | C*01:02:01e1 | | NK Cell | 28821 | 2971 | 4 | 562630 | 158336 | 4 |
| 111 |  | |  | | B Cell | 30865 | 3814 | 7 | 565437 | 24081 | 15 |
| HLA-A | A*11:01:01:01 | | A*03:01:01:01 | | CD4+ T Cell | 6205 | 508 | 7 | 580989 | 29471 | 15 |
| HLA-B | B*35:01:01:02 | | **B*51:01:01:01** | | CD8+ T Cell | 7950 | 978 | 7 | 765280 | 35990 | 15 |
| HLA-C | C*01:02:01e1 | | C*04:01:01:01 | | NK Cell | 14618 | 1823 | 7 | 831608 | 40602 | 15 |
| 112 |  | |  | | B Cell | 23792 | 4876 | 4 | 444896 | 51213 | 4 |
| HLA-A | A*68:02:01:01 | | A*02:01:01:01 | | CD4+ T Cell | 5161 | 2503 | 4 | 462751 | 34567 | 4 |
| HLA-B | B*15:07:01 | | **B*51:01:01:01** | | CD8+ T Cell | 5546 | 2772 | 4 | 549481 | 29808 | 4 |
| HLA-C | C*03:03:01e1 | | C*02:02:02:01 | | NK Cell | 8639 | 2296 | 4 | 464271 | 68611 | 4 |

| SAMPLE ID | Allele 1 | Allele 2 | Cell Type | Bw4 ABC Mean | | | | w6/32 ABC Mean | | |
| --- | --- | --- | --- | --- | --- | --- | --- | --- | --- | --- |
|  |  |  |  | Mean | SEM | | N | Mean | SEM | N |
| 119 |  |  | B Cell | 35711 | | 6594 | 4 | 513839 | 80343 | 4 |
| HLA-A | A*31:01:02:01 | A*11:01:01:01 | CD4+ T Cell | 4814 | | 818 | 4 | 419767 | 78497 | 4 |
| HLA-B | B*35:02:01 | **B*27:05:02** | CD8+ T Cell | 7119 | | 816 | 4 | 655265 | 189387 | 4 |
| HLA-C | C*02:02:02:01 | C*04:01:01:01 | NK Cell | 11104 | | 1317 | 4 | 618829 | 107306 | 4 |
| 121 |  |  | B Cell | 58116 | | 4788 | 8 | 539091 | 26583 | 16 |
| HLA-A | A*01:01:01:01 | - | CD4+ T Cell | 12104 | | 856 | 8 | 533203 | 18089 | 16 |
| HLA-B | B*08:01:01 | **B*27:05:02e1** | CD8+ T Cell | 18108 | | 1305 | 8 | 795882 | 26790 | 16 |
| HLA-C | C*02:07 | C*07:01:01:01 | NK Cell | 20363 | | 1477 | 8 | 761274 | 28861 | 16 |
| 126 |  |  | B Cell | 25458 | | 2456 | 12 | 502125 | 21520 | 12 |
| HLA-A | A*31:01:02:01 | A*29:01:01:01 | CD4+ T Cell | 9997 | | 1468 | 12 | 50503 | 26278 | 12 |
| HLA-B | B*07:05:01e1 | **B*51:01:01:01** | CD8+ T Cell | 14983 | | 1737 | 12 | 750702 | 40045 | 12 |
| HLA-C | C*15:02:01:01 | C*15:05:02 | NK Cell | 20461 | | 2119 | 12 | 641280 | 79411 | 12 |
| 128 |  |  | B Cell | 38913 | | 3317 | 7 | 693573 | 33279 | 15 |
| HLA-A | A*11:01:01:01 | A*02:01:01:01 | CD4+ T Cell | 11865 | | 1424 | 7 | 769691 | 51730 | 15 |
| HLA-B | B*15:01:01:01 | **B*44:02:01:01** | CD8+ T Cell | 12680 | | 1747 | 7 | 915760 | 56466 | 15 |
| HLA-C | C*03:03:01e1 | C*05:01:01:02 | NK Cell | 14047 | | 1294 | 7 | 695601 | 76937 | 15 |
| 130 |  |  | B Cell | 42280 | | 6050 | 4 | 533317 | 25972 | 10 |
| HLA-A | A*01:01:01:01 | A*30:01:01 | CD4+ T Cell | 9746 | | 2226 | 4 | 635477 | 25087 | 10 |
| HLA-B | B*08:01:01 | **B*13:02:01** | CD8+ T Cell | 17674 | | 2773 | 4 | 1094903 | 45510 | 10 |
| HLA-C | C*07:01:01:01 | C*06:02:01:01 | NK Cell | 19034 | | 3220 | 4 | 721555 | 31718 | 10 |
| 136 |  |  | B Cell | 48506 | | 2526 | 4 | 852880 | 84554 | 4 |
| HLA-A | A*11:01:01:01 | A*30:01:01 | CD4+ T Cell | 8199 | | 426 | 4 | 786137 | 44789 | 4 |
| HLA-B | B*35:01:01:02 | **B*13:02:01** | CD8+ T Cell | 15786 | | 1152 | 4 | 1267260 | 80903 | 4 |
| HLA-C | C*04:01:01:01 | C*06:02:01:01 | NK Cell | 11191 | | 502 | 4 | 1060278 | 83432 | 4 |
| 137 |  |  | B Cell | 54091 | | 4837 | 8 | 718262 | 36784 | 20 |
| HLA-A | A*01:01:01:01 | - | CD4+ T Cell | 8916 | | 1401 | 8 | 613849 | 39809 | 20 |
| HLA-B | B*08:01:01 | **B*37:01:01e1** | CD8+ T Cell | 17892 | | 1740 | 8 | 934235 | 58158 | 20 |
| HLA-C | C*07:01:01:01 | C*06:02:01:01 | NK Cell | 19652 | | 1058 | 8 | 620995 | 46623 | 20 |
| 141 |  |  | B Cell | 21527 | | 2063 | 4 | 504233 | 23486 | 20 |
| HLA-A | A*02:01:01:01 | A*03:01:01:01 | CD4+ T Cell | 5325 | | 285 | 4 | 493942 | 21004 | 20 |
| HLA-B | **B*35:01:01:02** | B*44:02:01:01 | CD8+ T Cell | 6369 | | 380 | 4 | 746468 | 28958 | 20 |
| HLA-C | C*05:01:01:02 | C*04:01:01:01 | NK Cell | 6656 | | 374 | 4 | 636857 | 27331 | 20 |
| 142 |  |  | B Cell | 63177 | | 3907 | 7 | 610495 | 17679 | 7 |
| HLA-A | A*02:01:01:01 | A*26:01:01:01 | CD4+ T Cell | 12451 | | 1367 | 7 | 541803 | 10760 | 7 |
| HLA-B | B*40:01:02 | **B*27:05:02e1** | CD8+ T Cell | 15453 | | 1386 | 7 | 690231 | 19128 | 7 |
| HLA-C | C*03:04:01:01 | C*01:02:01e1 | NK Cell | 10269 | | 929 | 7 | 496067 | 38891 | 7 |
| 143 |  |  | B Cell | 63132 | | 6357 | 4 | 754869 | 103176 | 6 |
| HLA-A | A*11:01:01:01 | A*03:01:01:01 | CD4+ T Cell | 14287 | | 815 | 4 | 600607 | 54010 | 6 |
| HLA-B | B*07:02:01 | **B*27:05:02** | CD8+ T Cell | 18737 | | 1361 | 4 | 824716 | 75767 | 6 |
| HLA-C | C*01:02:01e1 | C*07:02:01:03 | NK Cell | 21138 | | 2134 | 4 | 865303 | 85824 | 6 |

| SAMPLE ID | Allele 1 | Allele 2 | Cell Type | Bw4 ABC Mean | | | w6/32 ABC Mean | | | |
| --- | --- | --- | --- | --- | --- | --- | --- | --- | --- | --- |
|  |  |  |  | Mean | SEM | N | | Mean | SEM | N |
| 155 |  |  | B Cell | 34754 | 5250 | 8 | | 764098 | 79925 | 8 |
| HLA-A | A*26:01:01:01 | A*02:01:01:01 | CD4+ T Cell | 7406 | 1997 | 8 | | 706896 | 40122 | 8 |
| HLA-B | B*56:01:01:03 | **B*37:01:01e1** | CD8+ T Cell | 9409 | 2273 | 8 | | 877252 | 49231 | 8 |
| HLA-C | C*01:02:01e1 | C*06:02:01:01 | NK Cell | 16280 | 3098 | 8 | | 773175 | 73191 | 8 |
| 156 |  |  | B Cell | 69127 | 5462 | 15 | | 763282 | 29723 | 15 |
| HLA-A | A*01:01:01:01 | A*03:01:01:01 | CD4+ T Cell | 11521 | 1161 | 15 | | 508682 | 29444 | 15 |
| HLA-B | B*14:02:01:01 | **B*57:01:01** | CD8+ T Cell | 14700 | 1511 | 15 | | 746783 | 40144 | 15 |
| HLA-C | C*06:02:01:01 | C*08:02:01:01 | NK Cell | 20586 | 2542 | 15 | | 683006 | 34730 | 15 |
| 168 |  |  | B Cell | 23688 | 2615 | 9 | | 370210 | 21599 | 9 |
| HLA-A | A*02:01:01:01 | A*11:01:01:01 | CD4+ T Cell | 7388 | 1629 | 9 | | 509739 | 21106 | 9 |
| HLA-B | B*35:01:01:02 | **B*51:01:01:01** | CD8+ T Cell | 8106 | 1723 | 9 | | 614469 | 24297 | 9 |
| HLA-C | C*15:02:01:01 | C*04:01:01:01 | NK Cell | 6986 | 1658 | 9 | | 469479 | 20400 | 9 |
| 178 |  |  | B Cell | 49152 | 5835 | 10 | | 555081 | 21833 | 18 |
| HLA-A | A*02:01:01:01 | A*01:01:01:01 | CD4+ T Cell | 16243 | 1665 | 10 | | 639426 | 23368 | 18 |
| HLA-B | B*08:01:01 | **B*57:01:01** | CD8+ T Cell | 18678 | 2041 | 10 | | 787512 | 28761 | 18 |
| HLA-C | C*06:02:01:01 | C*07:01:01:01 | NK Cell | 13371 | 1485 | 10 | | 583752 | 28934 | 18 |
| 187 |  |  | B Cell | 27675 | 3796 | 4 | | 530435 | 38884 | 10 |
| HLA-A | A*02:01:01:01 | A*01:01:01:01 | CD4+ T Cell | 5688 | 790 | 4 | | 408892 | 37302 | 10 |
| HLA-B | **B*35:01:01:02** | B*44:02:01:01 | CD8+ T Cell | 5733 | 662 | 4 | | 508472 | 49710 | 10 |
| HLA-C | C*05:01:01:02 | C*04:01:01:05 | NK Cell | 6110 | 749 | 4 | | 496038 | 52006 | 10 |
| 194 |  |  | B Cell | 53987 | 4627 | 8 | | 428997 | 25830 | 16 |
| HLA-A | A*03:01:01:01 | A*01:01:01:01 | CD4+ T Cell | 13601 | 1701 | 8 | | 534632 | 31511 | 16 |
| HLA-B | B*07:02:01 | **B*57:01:01** | CD8+ T Cell | 17766 | 1912 | 8 | | 734497 | 43474 | 16 |
| HLA-C | C*07:02:01:03 | C*06:02:01:01 | NK Cell | 23311 | 3179 | 8 | | 649102 | 36294 | 16 |
| 198 |  |  | B Cell | 38983 | 5022 | 8 | | 579358 | 12549 | 18 |
| HLA-A | A*03:01:01:01 | A*01:01:01:01 | CD4+ T Cell | 10472 | 1890 | 8 | | 551187 | 16426 | 18 |
| HLA-B | B*08:01:01 | **B*57:01:01** | CD8+ T Cell | 14252 | 2320 | 8 | | 789102 | 25929 | 18 |
| HLA-C | C*06:02:01:01 | C*07:01:01:01 | NK Cell | 24008 | 3864 | 8 | | 757471 | 22645 | 18 |
| 202 |  |  | B Cell | 53042 | 4059 | 4 | | 630951 | 34115 | 4 |
| HLA-A | A*02:01:01:01 | A*30:01:01 | CD4+ T Cell | 9394 | 988 | 4 | | 671711 | 3595 | 4 |
| HLA-B | B*50:01:01 | **B*13:02:01** | CD8+ T Cell | 8842 | 958 | 4 | | 729133 | 14638 | 4 |
| HLA-C | C*06:02:01:01 | C*06:02:01:02 | NK Cell | 8083 | 1916 | 4 | | 650084 | 59254 | 4 |
