# Supplementary material for: Variations in HLA-B cell surface expression, half-life and extracellular antigen receptivity
Source: eLife. 2018 Jul 10;7:e34961. doi: 10.7554/eLife.34961 (PMC6039183; doi:10.7554/eLife.34961)
Supplement: Figure 6—source data 2. — Calculated HLA-Bw4 half-lives on lymphocytes from donors with relevant HLA-B genotypes indicated. The complete HLA class I genotypes of the donors are specified in Figure 6—source data 1. Mean half-life values are shown along with standard errors of mean half-life values (SEM) and the number of measurements (N; from separate blood collections) used for calculating the mean values. [file elife-34961-fig6-data2.docx]

**Figure 6 – Source Data 2 HLA-Bw4 stability on lymphocytes**

Calculated HLA-Bw4 half-lives on lymphocytes from donors with relevant HLA-B genotypes indicated. The complete HLA class I genotypes of the donors are specified in Figure 6-Source Data 1. Mean half-life values are shown along with standard errors of mean half-life values (SEM) and the number of measurements (N; from separate blood collections) used for calculating the mean values.

| Donor ID: | HLA-B Allele | B Cells | | | NK Cells | | | CD4 T Cells | | | CD8 T Cells | | |
| --- | --- | --- | --- | --- | --- | --- | --- | --- | --- | --- | --- | --- | --- |
|  |  | Mean | SEM | N | Mean | SEM | N | Mean | SEM | N | Mean | SEM | N |
| 121 | B*27:05 | 18.35 | 3.40 | 2 | 9.97 | 1.00 | 2 | 37.74 | 23.40 | 2 | 45.09 | 33.39 | 2 |
| 142 | B*27:05 | 21.10 | 6.28 | 3 | 11.08 | 0.51 | 3 | 23.47 | 4.46 | 3 | 19.68 | 5.88 | 3 |
| 8 | B*37:01 | 11.83 | 1.66 | 2 | 6.62 | 0.39 | 2 | 11.72 | 1.51 | 2 | 8.92 | 0.42 | 2 |
| 20 | B*37:01 | 12.59 | 0.91 | 2 | 6.82 | 0.18 | 2 | 13.90 | 1.28 | 2 | 10.52 | 0.85 | 2 |
| 155 | B*37:01 | 16.39 | 1.49 | 2 | 7.83 | 1.04 | 2 | 18.43 | 2.39 | 2 | 12.57 | 1.22 | 2 |
| 64 | B*44:02 | 22.30 | 6.24 | 3 | 13.29 | 4.32 | 3 | 17.24 | 4.89 | 3 | 14.43 | 2.01 | 3 |
| 80 | B*44:02 | 16.50 | 5.48 | 2 | 6.46 | 1.23 | 2 | 10.03 | 2.96 | 2 | 8.06 | 2.86 | 2 |
| 91 | B*44:02 | 16.20 | 0.24 | 2 | 8.88 | 1.66 | 2 | 13.84 | 2.54 | 2 | 13.55 | 1.16 | 2 |
| 128 | B*44:02 | 16.29 | 2.22 | 2 | 12.47 | 2.87 | 2 | 19.37 | 4.48 | 2 | 17.10 | 2.53 | 2 |
| 14 | B*51:01 | 14.50 | 2.03 | 2 | 6.81 | 0.05 | 2 | 13.94 | 0.68 | 2 | 11.99 | 1.58 | 2 |
| 62 | B*51:01 | 32.27 | 8.63 | 3 | 12.75 | 3.44 | 3 | 42.91 | 10.57 | 3 | 23.87 | 0.96 | 3 |
| 111 | B*51:01 | 21.83 | 5.56 | 2 | 12.22 | 3.01 | 2 | 22.15 | 4.10 | 2 | 18.90 | 3.22 | 2 |
| 126 | B*51:01 | 9.80 | 1.57 | 3 | 6.55 | 1.07 | 3 | 9.27 | 1.25 | 3 | 7.28 | 0.56 | 3 |
| 156 | B*57:01 | 43.10 | 12.57 | 3 | 20.92 | 5.05 | 4 | 51.70 | 17.95 | 4 | 26.81 | 6.81 | 4 |
| 194 | B*57:01 | 20.39 | 0.91 | 3 | 10.97 | 0.74 | 3 | 16.51 | 1.75 | 3 | 16.80 | 4.81 | 3 |
| 198 | B*57:01 | 17.75 | 1.88 | 2 | 8.81 | 0.77 | 2 | 13.53 | 1.33 | 2 | 17.03 | 2.40 | 2 |
