# Supplementary material for: Variations in HLA-B cell surface expression, half-life and extracellular antigen receptivity
Source: eLife. 2018 Jul 10;7:e34961. doi: 10.7554/eLife.34961 (PMC6039183; doi:10.7554/eLife.34961)
Supplement: Figure 7—source data 1. — HLA Bw4 genotypes of donors (full genotype in Figure 6—source data 1) and mean of ABC values measured with anti-Bw4 and W6/32 are shown for each lymphocyte or monocyte subset. Standard errors of the mean (SEM) values and the number of replicate measurements (N; with separate blood collections) are indicated. [file elife-34961-fig7-data1.docx]

**Figure 7 – Source Data 1: B Cell and Monocyte Bw4 ABC Values**

HLA Bw4 genotypes of donors (full genotype in Figure 6-source data 1) and mean of ABC values measured with anti-Bw4 and W6/32 are shown for each lymphocyte or monocyte subset. Standard errors of the mean (SEM) values and the number of replicate measurements (N; with separate blood collections) are indicated.

| SAMPLE ID | Allele 2 | Cell Type | Bw4 ABC Mean | | | w6/32 ABC Mean | | |
| --- | --- | --- | --- | --- | --- | --- | --- | --- |
|  |  |  | Mean | SEM | N | Mean | SEM | N |
| 14 |  |  |  |  |  |  |  |  |
| HLA-B | B*51:01:01:01 | B Cell | 42326 | 689 | 4 | 533611 | 46285 | 4 |
|  |  | Monocytes | 77091 | 2227 | 4 | 1347993 | 88356 | 4 |
| 64 |  |  |  |  |  |  |  |  |
| HLA-B | B*44: 02:01:01 | B Cell | 22077 | 372 | 4 | 461276 | 39325 | 4 |
|  |  | Monocytes | 17216 | 501 | 4 | 1065103 | 63458 | 4 |
| 94 |  |  |  |  |  |  |  |  |
| HLA-B | B*44: 02:01:01 | B Cell | 33087 | 793 | 4 | 972227 | 17357 | 4 |
|  |  | Monocytes | 15338 | 1467 | 4 | 1017014 | 65779 | 4 |
| 111 |  |  |  |  |  |  |  |  |
| HLA-B | B*51:01:01:01 | B Cell | 25188 | 1451 | 4 | 624174 | 21985 | 4 |
|  |  | Monocytes | 37445 | 2236 | 4 | 1275868 | 49362 | 4 |
| 126 |  |  |  |  |  |  |  |  |
| HLA-B | B*51:01:01:01 | B Cell | 19626 | 1796 | 4 | 475570 | 29353 | 4 |
|  |  | Monocytes | 57332 | 10509 | 4 | 1437062 | 97144 | 4 |
| 141 |  |  |  |  |  |  |  |  |
| HLA-B | B*44: 02:01:01 | B Cell | 21527 | 2063 | 4 | 551600 | 12450 | 4 |
|  |  | Monocytes | 12642 | 1621 | 4 | 1038441 | 43016 | 4 |
| 156 |  |  |  |  |  |  |  |  |
| HLA-B | B*57: 01:01 | B Cell | 48499 | 4242 | 6 | 751134 | 17540 | 6 |
|  |  | Monocytes | 40072 | 2250 | 6 | 1049272 | 45794 | 6 |
| 168 |  |  |  |  |  |  |  |  |
| HLA-B | B*51:01:01:01 | B Cell | 17779 | 995 | 4 | 362930 | 13268 | 4 |
|  |  | Monocytes | 36954 | 2972 | 4 | 1148943 | 103012 | 4 |
| 178 |  |  |  |  |  |  |  |  |
| HLA-B | B*57: 01:01 | B Cell | 36418 | 2312 | 6 | 575687 | 55310 | 6 |
|  |  | Monocytes | 42278 | 2263 | 6 | 1156690 | 67921 | 6 |
| 187 |  |  |  |  |  |  |  |  |
| HLA-B | B*44: 02:01:01 | B Cell | 27676 | 3796 | 4 | 499803 | 30958 | 4 |
|  |  | Monocytes | 9367 | 1809 | 4 | 667981 | 24147 | 4 |
| 198 |  |  |  |  |  |  |  |  |
| HLA-B | B*57: 01:01 | B Cell | 25883 | 383 | 4 | 553775 | 19902 | 4 |
|  |  | Monocytes | 46590 | 1645 | 4 | 1109658 | 57942 | 4 |
