# Supplementary material for: Variations in HLA-B cell surface expression, half-life and extracellular antigen receptivity
Source: eLife. 2018 Jul 10;7:e34961. doi: 10.7554/eLife.34961 (PMC6039183; doi:10.7554/eLife.34961)
Supplement: Figure 9—source data 1. — Top table: All genotyped donors. Bottom table: Blood donors whose samples were used for the data shown in Figures 1–7. [file elife-34961-fig9-data1.docx]

**Figure 9 - Source Data 1: Blood Donor demographics**

Top table: All genotyped donors

Bottom table: Blood donors whose samples were used for the data shown in Figures 1-7.

| Racial Categories | Ethnic Categories | | | |  |
| --- | --- | --- | --- | --- | --- |
|  | Not Hispanic or Latino | | Hispanic or Latino | | Total |
|  | Female | Male | Female | Male |  |
| American Indian/Alaska Native | 1 | 2 | 0 | 0 | 3 |
| Asian | 16 | 6 | 1 | 0 | 23 |
| Black or African American | 22 | 6 | 0 | 0 | 28 |
| White | 127 | 49 | 5 | 5 | 186 |
| Unknown/Not Reported | 1 | 3 | 0 | 0 | 4 |
| Total | 167 | 66 | 6 | 5 | **244** |
|  |  |  |  |  |  |
| American Indian/Alaska Native | 0 | 1 | 0 | 0 | 1 |
| Asian | 1 | 0 | 0 | 0 | 1 |
| Black or African American | 2 | 2 | 0 | 0 | 4 |
| White | 33 | 15 | 0 | 3 | 51 |
| Total | 36 | 18 | 0 | 3 | **57** |
