# Supplementary material for: Variations in HLA-B cell surface expression, half-life and extracellular antigen receptivity
Source: eLife. 2018 Jul 10;7:e34961. doi: 10.7554/eLife.34961 (PMC6039183; doi:10.7554/eLife.34961)
Supplement: Transparent reporting form [file elife-34961-transrepform.docx]

***eLife’s* transparent reporting form**

We encourage authors to provide detailed information *within their submission* to facilitate the interpretation and replication of experiments. Authors can upload supporting documentation to indicate the use of appropriate reporting guidelines for health-related research (see [EQUATOR Network](http://www.equator-network.org/%20)), life science research (see the [BioSharing Information Resource](https://biosharing.org/)), or the [ARRIVE guidelines](http://www.plosbiology.org/article/info:doi/10.1371/journal.pbio.1000412) for reporting work involving animal research. Where applicable, authors should refer to any relevant reporting standards documents in this form.

If you have any questions, please consult our Journal Policies and/or contact us: [editorial@elifesciences.org](mailto:editorial@elifesciences.org).

**Sample-size estimation**

- You should state whether an appropriate sample size was computed when the study was being designed
- You should state the statistical method of sample size computation and any required assumptions
- If no explicit power analysis was used, you should describe how you decided what sample (replicate) size (number) to use

Please outline where this information can be found within the submission (e.g., sections or figure legends), or explain why this information doesn’t apply to your submission:

For expression measurements, sample sizes for each included HLA-B allele were dependent on the representations of the relevant HLA-B allele within the donor pool of 244 healthy adults, combined with the absence of other defined cross-reactive HLA-B, HLA-A and HLA-C molecules. The details are outlined in the results section.

For Figure 1, as indicated in the results section, HLA-Bw6 alleles with at least three donors/allele who met the inclusion criteria were included in the expression measurements.

For Figure 6, as indicated in the results section, HLA-Bw4 alleles with at least four donors/allele who met the inclusion criteria were included in the expression measurements.

In other Figures, 3-4 donors per allele was taken to be an acceptable minimum sample size per allele for estimating allelic averages and statistically significant differences.

**Replicates**

- You should report how often each experiment was performed
- You should include a definition of biological versus technical replication
- The data obtained should be provided and sufficient information should be provided to indicate the number of independent biological and/or technical replicates
- If you encountered any outliers, you should describe how these were handled
- Criteria for exclusion/inclusion of data should be clearly stated
- High-throughput sequence data should be uploaded before submission, with a private link for reviewers provided (these are available from both GEO and ArrayExpress)

Please outline where this information can be found within the submission (e.g., sections or figure legends), or explain why this information doesn’t apply to your submission:

Data replicate information is specified in the Figure Legends and Source data sections.

As noted in the Methods section, half-life measurements were more variable with anti-Bw4 compared to anti-Bw6, and data for Bw4 half-lives are compiled only for donors with standard error of the mean half-life values less than 33% of the mean values (based on n ≥ 2 independent measurements) on all four measured lymphocyte populations.

**Statistical reporting**

- Statistical analysis methods should be described and justified
- Raw data should be presented in figures whenever informative to do so (typically when N per group is less than 10)
- For each experiment, you should identify the statistical tests used, exact values of N, definitions of center, methods of multiple test correction, and dispersion and precision measures (e.g., mean, median, SD, SEM, confidence intervals; and, for the major substantive results, a measure of effect size (e.g., Pearson's r, Cohen's d)
- Report exact p-values wherever possible alongside the summary statistics and 95% confidence intervals. These should be reported for all key questions and not only when the p-value is less than 0.05.

Please outline where this information can be found within the submission (e.g., sections or figure legends), or explain why this information doesn’t apply to your submission:

Statistical analyses and methods are discussed in the Figure legends and Methods sections. One-way analysis of variance (ANOVA) is used in most of the Figures, which is a valid method to assess statistically significant differences between multiple groups (in this case, groups of alleles)

Raw data are shown in the corresponding Source Data. N values are defined in the Source Data.

(For large datasets, or papers with a very large number of statistical tests, you may upload a single table file with tests, Ns, etc., with reference to sections in the manuscript.)

**Group allocation**

- Indicate how samples were allocated into experimental groups (in the case of clinical studies, please specify allocation to treatment method); if randomization was used, please also state if restricted randomization was applied
- Indicate if masking was used during group allocation, data collection and/or data analysis

Please outline where this information can be found within the submission (e.g., sections or figure legends), or explain why this information doesn’t apply to your submission:

Donors are allocated into allelic groups based on their HLA-Bw4 and/or HLA-Bw6 genotypes. The grouping is defined in the results section.

**Additional data files (“source data”)**

- We encourage you to upload relevant additional data files, such as numerical data that are represented as a graph in a figure, or as a summary table
- Where provided, these should be in the most useful format, and they can be uploaded as “Source data” files linked to a main figure or table
- Include model definition files including the full list of parameters used
- Include code used for data analysis (e.g., R, MatLab)
- Avoid stating that data files are “available upon request”

Please indicate the figures or tables for which source data files have been provided:

Figure 1-Source Data 1

Figure 2-Source Data 1

Figure 3-Source Data 1

Figure 3-Source Data 2

Figure 4-Source Data 1

Figure 5-Source Data 1

Figure 6-Source Data 1

Figure 6-Source Data 2

Figure 7-Source Data 1

Figure 9-Source Data 1
